# The synthesis of novel lanthanum hydroxyborate at extreme conditions

**DOI:** 10.3389/fchem.2023.1259000

**Published:** 2023-09-28

**Authors:** Olga Ibragimova, Lia Vaquero, Zain Hussein, Vadym Drozd, Stella Chariton, Vitali Prakapenka, Irina Chuvashova

**Affiliations:** ^1^ Department of Chemistry and Biochemistry, Florida International University, Miami, FL, United States; ^2^ Physics Department, Florida International University, Miami, FL, United States; ^3^ Department of Mechanical and Materials Engineering, Florida International University, Miami, FL, United States; ^4^ Center for Advanced Radiation Sources, The University of Chicago, Chicago, IL, United States

**Keywords:** high pressure, high temperature, synthesis, birefringence, lanthanum borate, rare-earth borates, new compound, diamond anvil cell

## Abstract

The novel structure of lanthanum hydroxyborate La_2_B_2_O_5_(OH)_2_ was synthesized by the reaction of partially hydrolyzed lanthanum and boron oxide in a diamond anvil cell under high-pressure/high-temperature (HPHT) conditions of 30 GPa and ∼2,400 K. The single-crystal X-ray structure determination of the lanthanum hydroxyborate revealed: $P\bar{3}c1$ , *a* = 6.555(2) Å, *c* = 17.485(8) Å, Z = 6, R_1_ = 0.056. The three-dimensional structure consists of discrete planar BO_3_ groups and three crystallographically different La ions: one is surrounded by 9, one by 10, and one by 12 oxygen anions. The band gap was estimated using *ab initio* calculations to be 4.64 eV at ambient pressure and 5.26 eV at 30 GPa. The current work describes the novel HPHT lanthanum hydroxyborate with potential application as a deep-ultraviolet birefringent material.

## 1 Introduction

In recent decades, borates have garnered significant interest as versatile materials due to their varied crystal structures, impressive linear and nonlinear optical (NLO) properties, and favorable machining characteristics (Chen et al., 1995; Wu et al., 1996; Chénais et al., 2002; Jia et al., 2017; Zou and Ok, 2020; Guo et al., 2022). Notably, considerable research efforts have been devoted to the development of novel borate-based birefringent materials for potential application in the deep-ultraviolet (DUV) region (longer wavelength portion of UV-C; 190 nm ≤ λ_0_ ≤ 280 nm; 4.43 eV ≤ E_0_ ≤ 6.53 eV) (Zhang et al., 2020).

Despite the different principles in crystal symmetries of NLO and birefringent materials, sufficiently large optical anisotropy plays a crucial role in the applications for both types (Guo et al., 2022). It is widely recognized that the small double refraction of NLO crystals limits their ability to achieve the shortest phase-matching wavelength, thereby negatively affecting potential applications in the DUV region (Jiang et al., 2015). Birefringent materials play an important role in modulating light polarization in the optical communication and laser industries (Li and Ma, 2012). Several commercially available crystals like calcite (Ghosh, 1999), YVO_4_ (DeShazer, 2002), α-BaB_2_O_4_ (Guoqing et al., 1998; Solntsev et al., 2002), and MgF_2_ (Dodge, 1984) have found utility in the optical spectrum spanning from the infrared (IR) to the UV range with wavelengths below 400 nm. However, these four compounds are not suitable for application in the DUV region because:

• The low transmittance in the DUV range of α-BaB_2_O_4_, calcite, and YVO_4_ restricts their application in shorter-wavelength regions;
• An extremely small birefringence (Δn = 0.0128 at 235.7 nm) limits the application of MgF_2_ in the DUV region (Scott, 1962; Dodge, 1984).

Consequently, an ideal crystal must possess high transmittance in the DUV range, large birefringence (Δn > 0.08) (Gong et al., 2020), and a short UV cutoff edge (the smaller the better) (Guo et al., 2022). Today, the scarcity of such materials meeting these criteria underscores the importance of exploring novel DUV crystals with substantial birefringence.

In terms of borates, there are three basic groups that form unique structures: linear BO_2_, trigonal-planar BO_3_ and tetrahedral BO_4_, which occur as discrete oxyanions or polymerize to form finite clusters, chains, sheets, and frameworks. Anionic group theory gives the understanding of structure-property relationships. The key factor that affects optical properties is if the anionic unit is capable to produce a wide band gap, high polarizability anisotropy, and hyperpolarizability (Huang et al., 2021; Mutailipu et al., 2021). The triangle π-conjugated BO_3_ group has the largest hyperpolarizability (β_max_ = 10.80) among BO_2_ (β_max_ = 0) and BO_4_ (β_max_ = 3.61), and a good relation between the other two parameters: band gap (E_g_ = 8.48 eV) and polarizability anisotropy (*δ* = 7.01) (Mutailipu et al., 2021). The birefringence originates from the dependence of dipole oscillators and refractive index, and it can be considered as a result of the functional unit`s arrangement (Wang F. et al., 2021). Therefore, the solution for the large optical anisotropy lies in metal cations and the type of functional anion (Jin et al., 2021). The metal cations can form MO_n_ polyhedra with large deformability and thereby increase the optical anisotropy (Wu et al., 2019). But with the positive influence on birefringence, there is a negative effect on DUV transmittance: the crystals with big metal cations have a smaller band gap and as a consequence may hardly transmit in the DUV spectral region. The birefringence is not sensitive to the exact orientation of the BO_3_ groups because it is almost optically isotropic within the BO_3_ plane (Chen et al., 2012). It was shown that all borates crystal structures with only triangle borate groups could be divided into three structural types according to the arrangement of these groups: 1) the BO_3_ planes arranged in a nearly coplanar pattern, 2) in a coaxial pattern, 3) and in the other inclined patterns (Jiang et al., 2015). The crystals with coplanar arrangement generally have the largest birefringence, while those with disordered patterns have the smallest (Jiang et al., 2014; Lin et al., 2014).

Along with alkali and transition metals borates, rare-earth (RE) borates (and lanthanum borates in particular) have raised significant interest due to their stable physical and chemical properties, wide bandwidth, and good light transmittance. The system La-B-O is represented by several compositionally different borates: λ-LaBO_3_ (Sari et al., 2017), high-temperature modification H-LaBO_3_, LaB_3_O_6_ (ambient pressure), and its two high-pressure modifications γ-LaB_3_O_6_ (Emme et al., 2004) and δ-LaB_3_O_6_ (Heymann et al., 2006), La_4_B_14_O_27_ (Nikelski et al., 2008), La_26_O_27_(BO_3_)_8_ (Lin et al., 1996), La_4_B_10_O_21_ (isotypic to Pr_4_B_10_O_21_) (Hinteregger et al., 2012) and La_2_B_8_O_15_ (isotypic to Ce_2_B_8_O_15_) (Glätzle et al., 2016). Except for the high-pressure modifications (γ-LaB_3_O_6_, δ-LaB_3_O_6,_ and La_4_B_10_O_21_), all known lanthanum borates are synthesized by heating stoichiometric mixtures of La_2_O_3_ and B_2_O_3_ under ambient pressure conditions. However, this simple synthesis fails to produce pure crystalline phases (Shmyt’ko et al., 2013). Hence, HPHT synthesis serves as a viable alternative. There is a common trend in high-pressure borates where the boron atoms tend to prefer four-fold coordination as pressure increases. Typically, trigonal-planar BO_3_ groups transform into tetrahedral BO_4_ groups beyond 10 GPa pressure (Hinteregger et al., 2012). However, a few compounds are known to contain trigonal-planar BO_3_ groups beyond this pressure threshold, for example, in Ho_31_O_27_(BO_3_)_3_(BO_4_)_6_ (Hering et al., 2010).

In the present study, we have applied methods of single-crystal X-ray diffraction in laser-heated diamond anvil cell (DAC) to synthesize lanthanum hydroxyborate La_2_B_2_O_5_(OH)_2_ for the first time and characterize its crystal structure. We performed *ab initio* calculations of the band gap and compared both: the band gap and its structural properties with literature. The structural organization of La_2_B_2_O_5_(OH)_2_ allows us to propose that the lanthanum borates with isolated BO_3_ groups can be used as a potential DUV birefringent material.

## 2 Materials and methods

### 2.1 Sample preparation

For the synthesis, we used a non-stoichiometric mixture of partially hydrolyzed La (a piece of lanthanum 99.9% purity from Fisher Scientific left on humid air for 3 days) and B_2_O_3_ (99.999% purity, purchased from Fisher Scientific), finely grounded and pre-pressed into pellets between two diamonds. The pre-pressed pellet of size 10*15*5 μm^3^ was loaded in the symmetric DAC (Symm100 by Almax EasyLab) equipped with Boehler–Almax diamonds with a 200 μm culet size. Rhenium gasket was pre-indented to the thickness of 26 μm, and a hole with the diameter of about 110 μm was drilled with a laser-drilling system at GeoSoilEnviroCARS (GSECARS), Advanced Photon Source (APS), Argonne National Laboratory (ANL). A 4 μm piece of gold and a 15 μm ruby ball (DACtools, LLC) were used as pressure calibrants (Ye et al., 2018). Neon was loaded using the COMPRES/GSECARS gas loading system available at APS (Rivers et al., 2008) and served as a pressure-transmitting medium.

### 2.2 Laser-heating experiments

Double-sided laser-heating experiments coupled with X-ray diffraction (XRD) measurements were conducted at the 13ID-D beamline at GSECARS of the APS, ANL. The laser heating system at the beamline is equipped with two 1,064 nm wavelength infrared lasers that produce a flat-top spot size of around 10 µm in diameter (full width at half-maximum) (Goncharov et al., 2010). We used double-sided laser heating in the burst mode with a heating duration of 1 or 3 s, collecting XRD of the sample during and after heating. The surface temperatures of both sides were measured by the standard spectroradiometry method (Heinz and Jeanloz, 1987) using an IsoPlane SCT 320 spectrometer with a PI-MAX3 1024i ICCD camera from Princeton Instruments.

The DAC was compressed stepwise to a maximum pressure of 30(1) GPa and laser-heated at 3.3(5) and 13.3(5) GPa to a maximum temperature of 2,285(325) and 2,430(130) K, respectively. After each heating cycle, a detailed X-ray diffraction map was collected around the heated spot in order to determine the occurrence of the reaction and the phase composition. At each pressure point, single crystal X-ray diffraction images were collected in a few spots with decent quality for phase composition determination.

### 2.3 X-ray data analysis

The crystal structures of the sample in the DAC were investigated through *in situ* XRD measurements using synchrotron radiation performed at the 13ID-D beamline of the APS, ANL. A monochromatic X-ray beam of 42 keV (*λ* = 0.2952 Å) was used to collect powder and single crystal data on a Pilatus 1M CdTe large area detector.

#### 2.3.1 Powder diffraction

Powder collection was performed as a single image in the omega scanning range of ±20° with an exposure time of 40 s. Detector parameters were calibrated using LaB_6_.

The data for powder analysis was prepared and converted into the required file extension using Dioptas software (Prescher and Prakapenka, 2015). Powder XRD data analysis consisted of structure refinement by the Le Bail method using the GSAS II software (Toby and Von Dreele, 2013).

#### 2.3.2 Single crystal diffraction

Single crystal collection included 120 frames in the omega scanning range of ±34° with a step of 0.5° and an exposure time of 1 s per step. Sample-to-detector distance, coordinates of the beam center, tilt angle, and tilt plane rotation angle of the detector images were calibrated using an orthoenstatite crystal.

Single-crystal XRD data (unit cell determination, integration of the reflection intensities, absorption corrections) were processed using CrysAlisPro software (Rigaku Oxford Diffraction, 2019). For the search of the domains during data reduction, the DAFi software was used (Aslandukov et al., 2022). The structure was determined by SHELXT (Sheldrick, 2015), a structure solution package that uses the method of intrinsic phasing. The crystal structure was refined against *F* ^2^ on all data by full-matrix least-squares using SHELXL software (Sheldrick, 2015). SHELXT and SHELXL were implemented in the Jana2006 software package (Petříček et al., 2014). Crystal structure visualization was made using VESTA (Momma and Izumi, 2011) and Diamond software (Putz and Brandenburg, 1999).

Because more than 50% of the diffraction reflections were blocked by the body of the diamond anvil cell, the data sets of the reflections were incomplete. To improve the data/parameter ratio, only the atomic thermal parameters of lanthanum and oxygen were refined using anisotropic approximation, leading to an R_1_ value of 5.6%. Due to the presence of high-Z lanthanum atoms, the residual electron density peaks were on the order of 2–6 e/Å^3^, which is comparable with the number of electrons in boron and oxygen atoms. Assignment of the residual density peaks to boron or oxygen atoms did not improve the final R-values, and therefore, the high residuals likely originate from the incompleteness of the XRD data sets. The typical data/parameter ratios were on the order of 10–15. The detailed summary of the crystal structure refinement, along with unit cell parameters, atomic coordinates, and atomic displacement parameters, is shown in Table 1 and Supplementary Tables S1, S2.

**TABLE 1:** Crystal data and structure refinement parameters for La_2_B_2_O_5_(OH)_2_.

Empirical formula	La_2_B_2_O_5_(OH)_2_
Crystal system	Trigonal
Space group	$P\bar{3}c$ 1
Space group number	165
a (Å)	6.556(2)
c (Å)	17.485(9)
V (Å^3^)	650.8(5)
Z	6
R_int_	0.06
R_1_	0.056
GOF (obs)	1.82
GOF (all)	1.42
# reflections (total)	764
# of refinable parameters	46

### 2.4 Scanning electron microscopy

The composition of the sample mixture prior to loading in the DAC was analyzed by means of scanning electron microscopy using the JOEL JSM-IT500HR scanning electron microscope (JEOL USA, Inc., Peabody, MA, United States). The chemical composition was checked at 15 kV using energy-dispersive X-ray spectroscopy (EDS) of QUANTAX EDS System with XFlash 6160 detector (Bruker Nano GmbH, Berlin, Germany). No impurities or traces of any other elements were found. The detailed summary is shown in Supplementary Figure S1.

### 2.5 Band gap calculations

Density functional theory (DFT) calculations were performed using Vienna *ab initio* simulation package (VASP 6.3) (Kresse and Furthmüller, 1996a; Kresse and Furthmüller, 1996b). Local Density Approximation (LDA) and Generalized Gradient Approximation (GGA) were used to describe exchange and correlation effects. Electron wave functions were expanded by plane wave with a cutoff energy of 520 eV. Monkhorst-Pack (Monkhorst and Pack, 1976) *k*-point grids were set as 12 × 12 × 4 for the structure relaxation and 24 × 24 × 8 for the electronic structure calculations. Atomic relaxation was performed until the change in the electronic and ionic steps were less than 10^−6^ and 10^−5^ eV, respectively. VASPKIT package (Wang V. et al., 2021) was used to extract and analyze the VASP raw output files. Calculations were performed at ambient pressure and external pressure of 30.0 GPa.

## 3 Results

The synthesis of new lanthanum hydroxyborate was performed from the mixture of partially hydrolyzed lanthanum powder and boron oxide. The initial mixture has irregular composition (insert in Supplementary Figure S1). As lanthanum can oxidize on air to either oxide, hydroxide, carbonate, or hydroxycarbonate depending on the conditions and humidity, we performed EDS analysis to confirm the composition of starting mixture. The brief inspection of the EDS spectrum of the initial mixture shows that the sample is composed of the elements La, B, and O. We used carbon tape as a sample mount which may account for the carbon present in the spectra.

**FIGURE 1:**
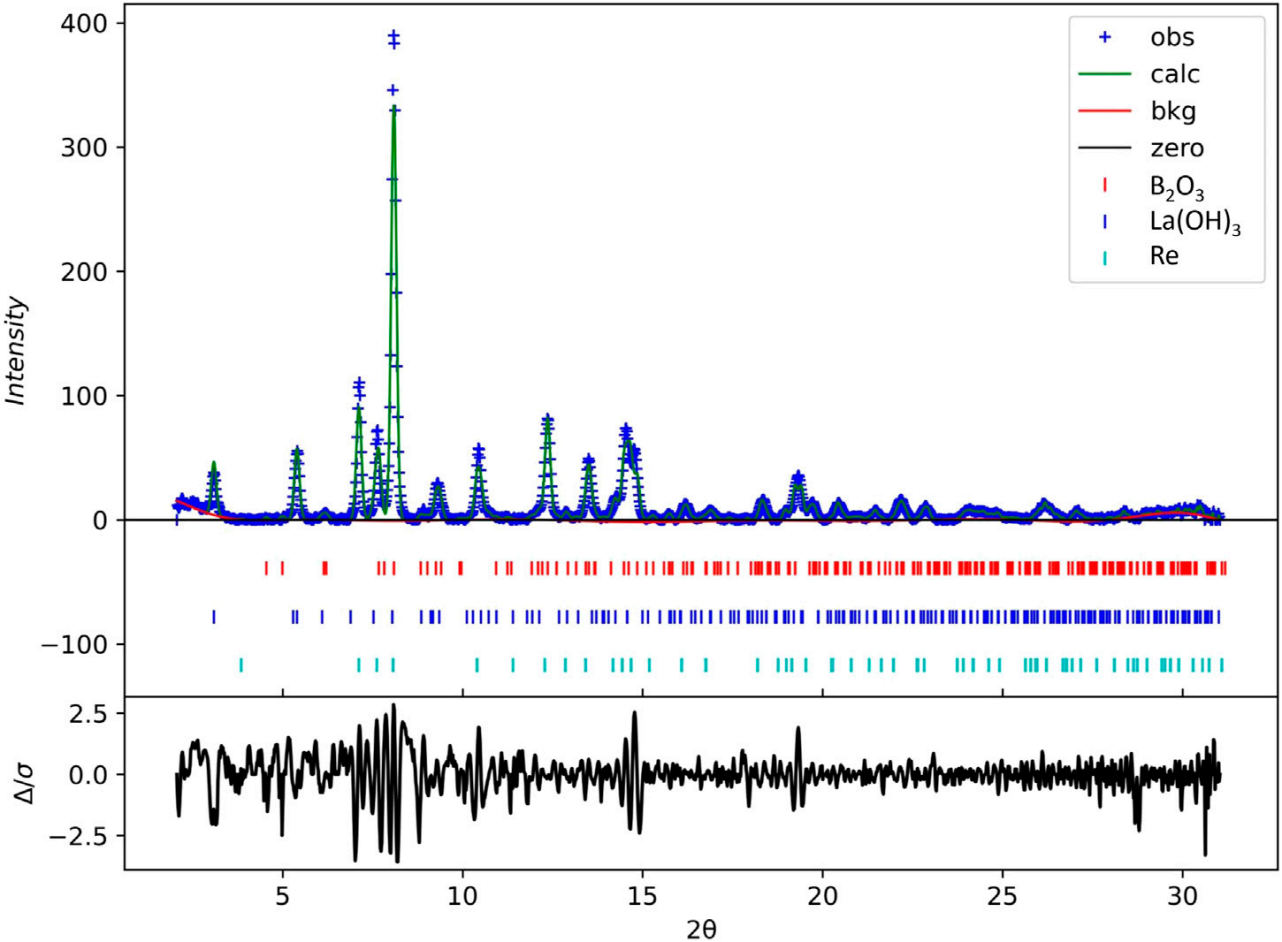
Powder XRD pattern of the mixture prior to heating at 3.3(5) GPa: blue crosses are experimental data from this study, the green line corresponds to the calculated curve, the red one represents the background, and the black one shows the difference between observed and calculated curves. The ticks represent the different phases: red is B_2_O_3_ (ICSD 16021), blue is La(OH)_3_ (ICSD 239411), and teal one is Re (ICSD 291898).

To confirm the composition of the initial mixture, we performed powder XRD analysis at the pressure of 3.3(5) GPa. The powder pattern can be found in Figure 1. There are characteristic peaks of La(OH)_3_, B_2_O_3_, and Re. The unit cell parameters for La(OH)_3_ (*P*6_3_ */m*) are: a = 6.506(4) Å, c = 3.8673(7) Å, V = 141.75(12) Å^3^; for B_2_O_3_ (*P*3_1_) are: a = 4.3789(11) Å, c = 8.2888(16) Å, V = 137.64(5) Å^3^; and for Re (*P*6_3_ */mmc*) are: a = 2.716(7) Å, c = 4.396(30) Å, V = 28.09(20) Å^3^. The R-factor is equal to 0.6%. Thus, the source of lanthanum in the system was lanthanum hydroxide. Our sample is very close to the gasket; therefore, the presence of rhenium is not surprising. No characteristic peaks of other compounds exist, such as lanthanum carbonate, lanthanum hydroxycarbonate, or lanthanum oxycarbonate. We did not observe the characteristic peaks of various lanthanum borate phases either. As a result, we can rule out the possibility of a reaction occurring during compression at 3.3(5) GPa without heating.

Laser heating up to 2,285(325) K at 3.3(5) GPa led to a pressure jump to 6.6(5) GPa, however, the reaction did not occur. Data collection at 13.3(5) GPa before and after heating to 2,430(130) K did not produce good-quality crystalline samples either. Fortunately, the diffraction pattern after increasing pressure up to 30(1) GPa confirmed the chemical reaction by forming a new, previously unknown structure.

**FIGURE 2:**
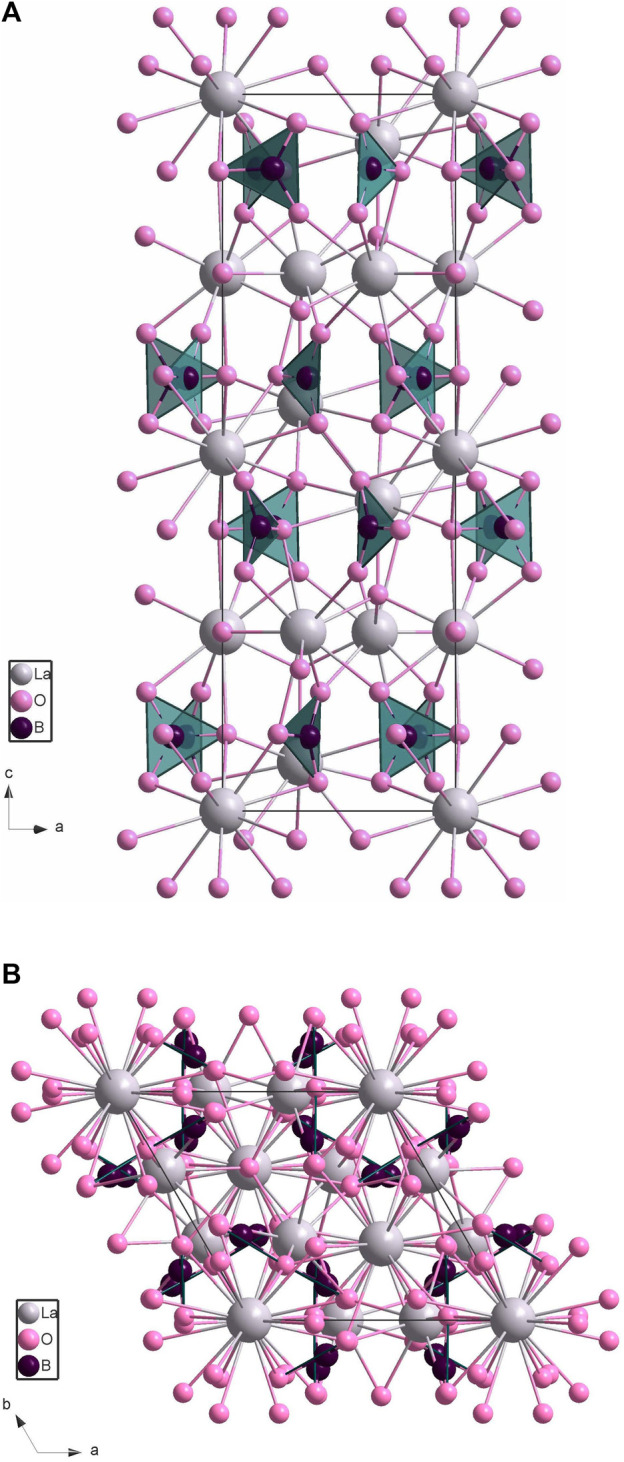
Crystal structure of La_2_B_2_O_5_(OH)_2_ (hydrogen atoms are not shown): **(A)** along *a* axis, **(B)** along *c* axis. Lanthanum atoms are shown in grey, oxygens are pink, and boron atoms are in the center of each planar triangle and are shown in dark purple.

By employing single crystal diffraction analysis, the new compound crystallizes in the centrosymmetric trigonal space group $P\bar{3}c1$ (no. 165) with lattice parameters *a* = 6.555(2) Å, *c* = 17.485(8) Å (Table 1). It consists of a dense, three-dimensional network (Figure 2) composed of planar triangles of BO_3_ connected with three crystallographically independent lanthanum ions. The lanthanum atoms possess different coordination spheres (Supplementary Figure S2): La01 is coordinated by 10 oxygen atoms, La02 is coordinated by 12 oxygen atoms, and La03 is coordinated by 9 oxygen atoms. The distances La-O are in the range of 2.27(2)–2.614(12) Å. The B-O bond lengths are in the range of 1.26(2)–1.321(15) Å (for all bond lengths in the structure, see Supplementary Table S3). These bond distances are slightly smaller than those published for alkali- and rare-earth borates which can be explained by the higher pressure reached during this experiment.

As the amount of water absorbed by the lanthanum is unknown, for the structure solution, we used only three elements: La, B, and O. According to the structure refinement, our formula ended up as La_2_B_2_O_7_. To achieve charge balance, instead of two oxygen atoms, there should be two hydroxyl groups. This agrees well with our XRD data of the starting compositions. Therefore, the new borate has a composition of La_2_B_2_O_5_(OH)_2_.

**FIGURE 3:**
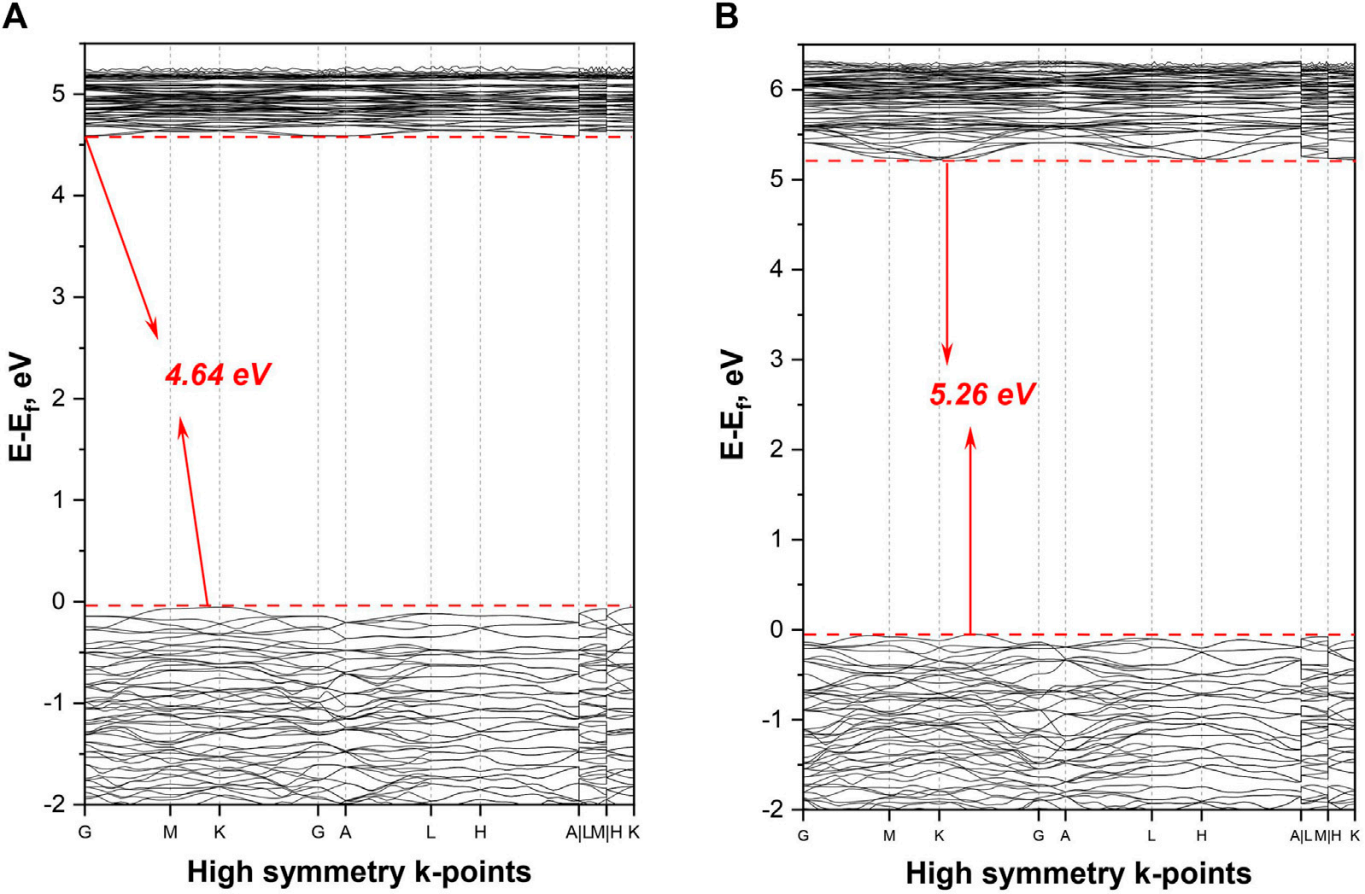
The electronic band structure calculated by using LDA method: **(A)** for ambient pressure, and **(B)** for 30 GPa.

The band gap calculated by DFT method is 3.40 eV (GGA)/4.64 eV (LDA) at ambient pressure and 5.31 eV (GGA)/5.26 eV (LDA) at 30 GPa pressure (Figure 3 for LDA and Supplementary Figure S4 for GGA). These values correspond to the short cutoff edge in the range from 365 to 233 nm. Commonly, the GGA and LDA method-based calculations tend to slightly underestimate the band gap, therefore the real value might be slightly higher and, consequently, the cutoff edge smaller (Bagayoko, 2014; Baiheti et al., 2021).

## 4 Discussion

The starting mixture for the reaction consisted of La(OH)_3_ and B_2_O_3_. During laser heating up to 2,430(130) K, the hydroxide partially decomposed to La_2_O_3_ and H_2_O without subsequent melting of La_2_O_3_ [T_m (amb)_ = 2588 K (Grundy et al., 2000)], while boron oxide melted [T_m (8 GPa)_ ∼ 1,800 K (Solozhenko et al., 2015)]. The behavior of B_2_O_3_ melt is similar to SiO_2_, and it readily dissolves metallic oxides with the formation of borates (Huppertz, 2011), so the lanthanum borate was synthesized by dissolving La_2_O_3_ and La(OH)_3_ in B_2_O_3_ with subsequent reaction.

**FIGURE 4:**
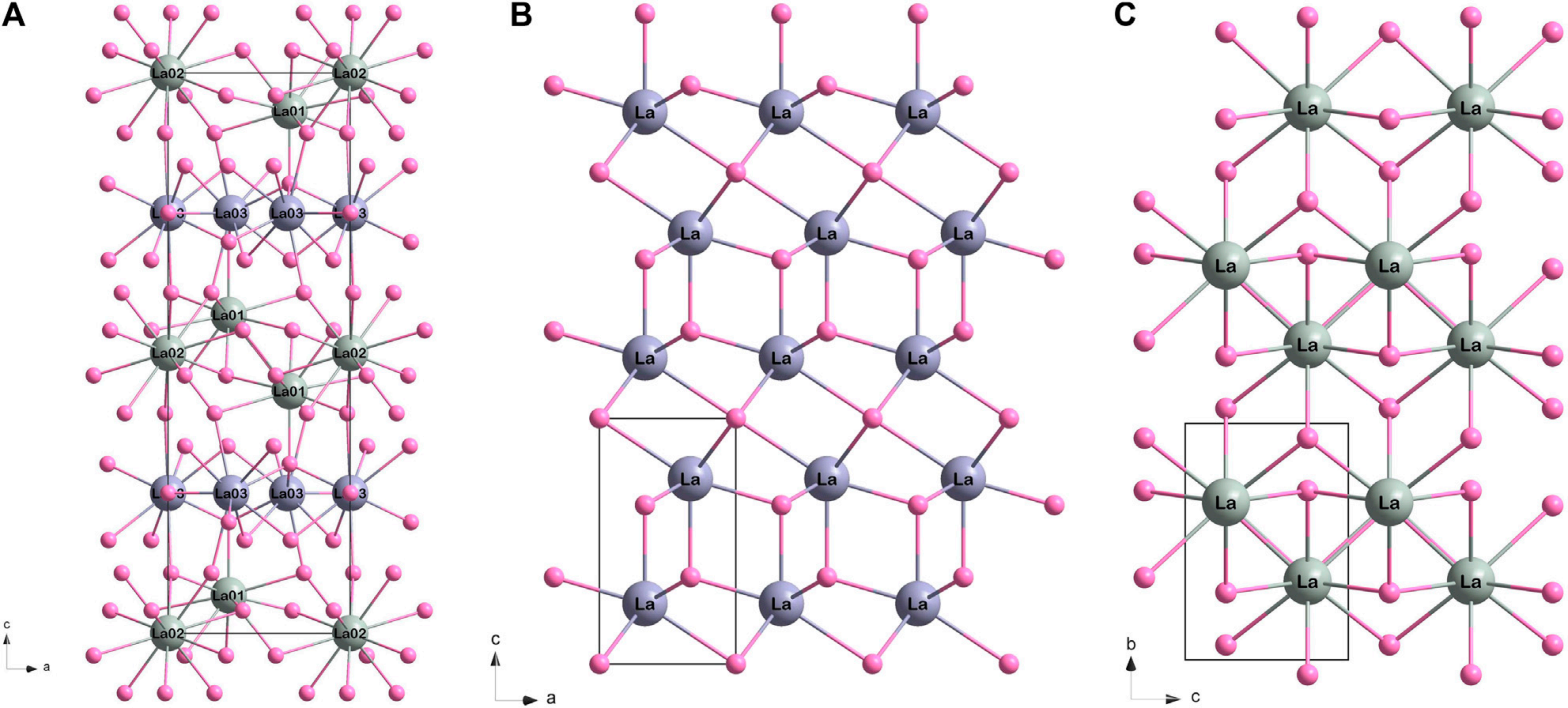
The comparison of La-O frame (hydrogen and boron atoms are not shown) for: **(A)** La_2_B_2_O_5_(OH)_2_ (present work), **(B)** La_2_O_3_ (ICSD 56771), and **(C)** La(OH)_3_ (ICSD 239411). For convenience, the lanthanum atoms with different pattern are shown in different shades of grey: for La_2_O_3_ pattern in violetish grey, for La(OH)_3_ pattern in greenish grey.

The structure of La_2_B_2_O_5_(OH)_2_ is based on La-O layers identical to those in the A-type La_2_O_3_ structure ( $P\bar{3}m$ ) and La(OH)_3_ (P6_3_ */m*) (Figure 4). During HP synthesis, the lanthanum-oxygen layers of La(OH)_3_ were integrated between similar layers of La_2_O_3_. The oxide layers are rotated by 180° relative to each other along the *c* axis. The coordination numbers for La in La(OH)_3_ frame increase from 9 to 10 and 12 [La01 and La02 in La_2_B_2_O_5_(OH)_2_]; while for La in La_2_O_3_ pattern—from 7 to 9 [La03 in La_2_B_2_O_5_(OH)_2_]. Boron atoms have three-fold coordination and are located in the cavities between La01/02-O and La03-O layers. The positional disorder of the BO_3_ groups is unusual for known RE borates and has never been described before. In terms of a three-fold coordinated boron atom, in RE borates BO_3_ group can form either linked chains [for example, in α-Sm(BO_3_)_2_ (Fuchs et al., 2020a) or in LaB_2_O_4_F (Hinteregger et al., 2013b)], or isolated triangles [for instance, in NdBO_3_ (Müller-Bunz et al., 2003) or in Dy_5_(BO_3_)_2_F_9_ (Hinteregger et al., 2013a)]. The arrangement of boron groups in most cases is coplanar (parallel to one plane) or slightly distorted. In La_2_B_2_O_5_(OH)_2_ boron groups have a coaxial arrangement: each BO_3_ triangle occupies one of three possible positions along C_3_ axis and lies in the glade *n* plane (Supplementary Figure S5). BO_3_ group exhibits C_2_ symmetry as it represents an isosceles triangle.

**FIGURE 5:**
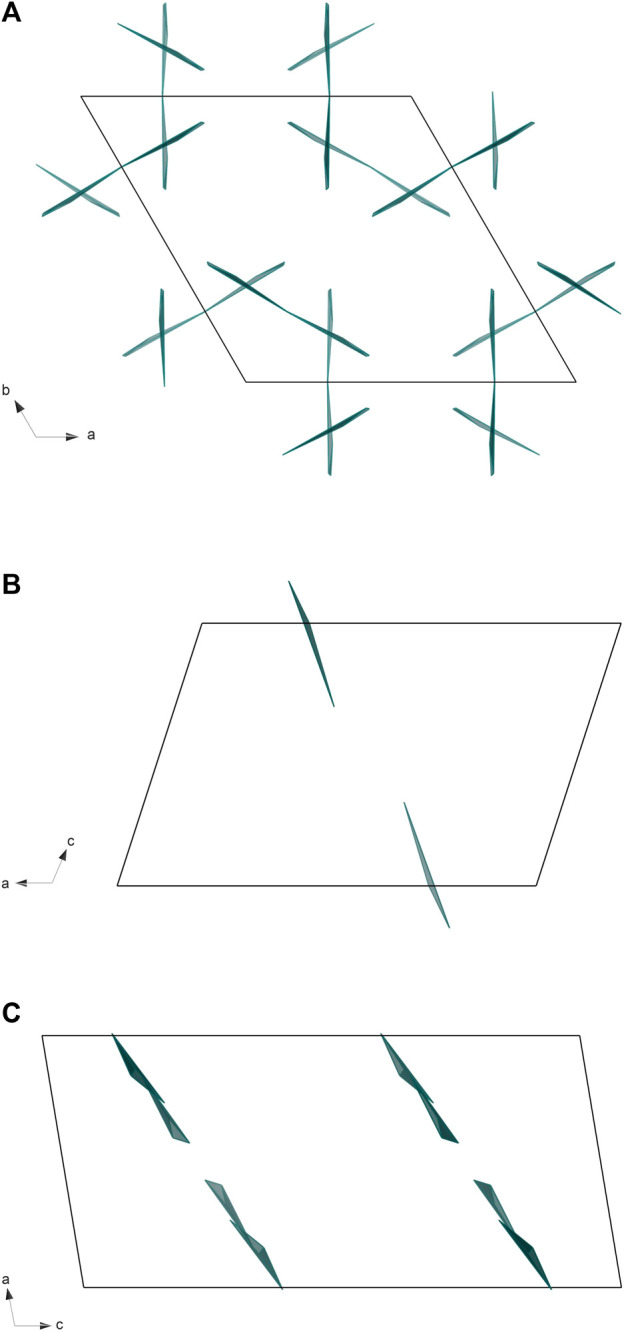
The comparison of BO_3_ polyhedra arrangement for: **(A)** La_2_B_2_O_5_(OH)_2_ (present work), **(B)** H-LaBO_3_ (ICSD 15383), and **(C)** La_26_(BO_3_)_8_O_27_ (ICSD 82308). For convenience, only BO_3_ polyhedra are shown.

**FIGURE 6:**
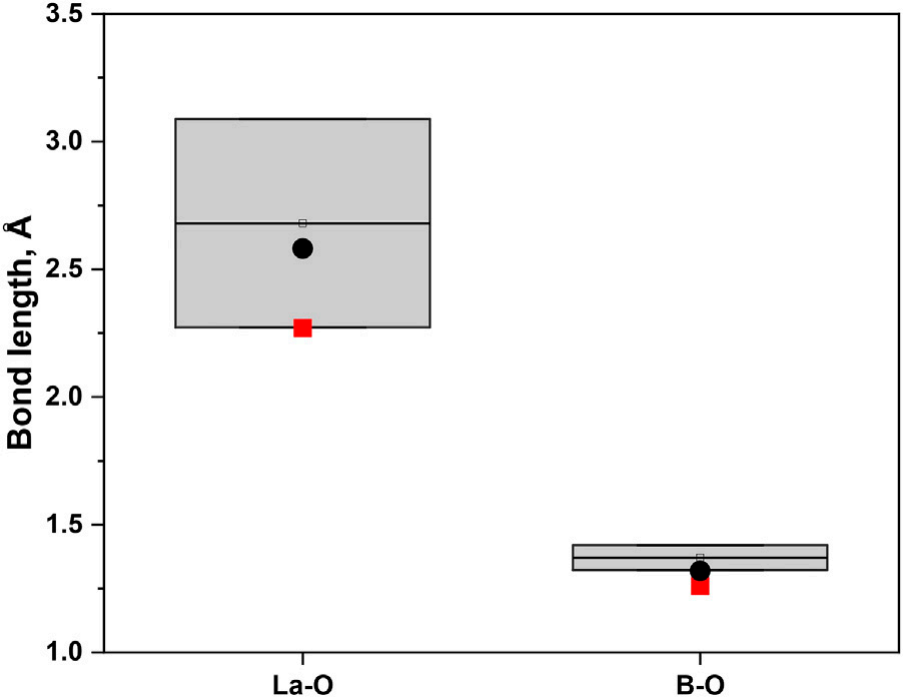
The comparison of the bond distances for La-O and B-O (in trigonal planar orientation) from publications (Böhlhoff et al., 1971; Lin et al., 1996; Emme et al., 2004; Hinteregger et al., 2012) and for La_2_B_2_O_5_(OH)_2_ (present work). The ranges from the literature are shown in grey bars, the red squares are corresponding to the smallest value of the bond length of the La_2_B_2_O_5_(OH)_2_, while the black dots are representing the biggest value.

There are known structures of RE hydroxyborates, for example, high-pressure La_3_B_6_O_13_(OH) (Fuchs et al., 2020b), and LaB_2_O_4_(OH) (Bashir et al., 2017). However, both are composed of edge-sharing BO_4_ tetrahedra which distinguishes them from the newly synthesized La_2_B_2_O_5_(OH)_2_. We found only two lanthanum borates with similar structure: H-LaBO_3_ (Böhlhoff et al., 1971) and La_26_(BO_3_)_8_O_27_ (Lin et al., 1996). Both these structures consist of repeated La-O and BO_3_ patterns: the orientation of planar BO_3_ groups in H-LaBO_3_ is coplanar, while in La_26_(BO_3_)_8_O_27_ the BO_3_ groups have a slightly distorted arrangement (Figure 5). The bond distances in La_2_B_2_O_5_(OH)_2_ correspond well to typical bond lengths in lanthanum borates (Figure 6). For example, in comparison with La_26_(BO_3_)_8_O_27_ at ambient pressure where the bond distances for La-O are in the range 2.272(10)–3.047(9) Å, and for B-O are in the range 1.34(2)–1.42(2) Å (Lin et al., 1996), the newly synthesized La_2_B_2_O_5_(OH)_2_ at 30(1) GPa have the bond lengths 2.27(2)–2.612(14) Å and 1.26(2)–1.319(15) Å for La-O and B-O, respectively.

While the borates with parallel arrangement of planar BO_3_ groups have the largest birefringence (Jiang et al., 2015), the situation with coaxial BO_3_ arrangement is slightly different. The value for optical anisotropy depends not only on BO_3_ pattern, but also on the detailed orientation and spatial density of the planar BO_3_ groups (Jiang et al., 2015). Additionally, the presence of hydroxyl anions linked with isolated triangular boron groups may enlarge the birefringence by delocalization of π electrons (Jin et al., 2021). Our calculated band gap values for ambient [3.40 eV (GGA)/4.64 eV (LDA)] and high pressure [5.31 eV (GGA)/5.26 eV (LDA)] are typical for lanthanum borates with three-fold coordinated boron. For example, the calculated (GGA) and experimental band gap at ambient conditions for LaBO_3_ with two-dimensional BO_3_ layers are 4.81 and 5.23 eV (Sha et al., 2021) respectively. As mentioned earlier, the presence of rare-earth elements may positively affect the birefringence but at the same time decreases the band gap. As we are looking at the combination of factors, there is a good probability that synthesized La_2_B_2_O_5_(OH)_2_ has high birefringence with the cutoff edge short enough to transmit in the longer wavelength portion of UV-C.

## 5 Conclusion

During the HPHT synthesis at 30(1) GPa and 2,430(130) K, we were able to obtain a new structure of lanthanum hydroxyborate [La_2_B_2_O_5_(OH)_2_] with planar BO_3_ groups. In contrast with the most known RE borates (including high-pressure structures), La_2_B_2_O_5_(OH)_2_ does not form a B-O framework and does not have BO_4_ tetrahedra: trigonal-planar BO_3_ groups occupy the cavities between La-O layers and create the coaxial pattern. The estimated band gap for lanthanum hydroxyborate is 4.64 eV (for ambient pressure) and 5.26 eV (for 30 GPa), which fits well into the acceptable range for DUV transparency. The structural organization of La_2_B_2_O_5_(OH)_2_ and its electronic properties allows us to propose that this class of lanthanum borates can be used as a potential DUV birefringent material.

## Data Availability

The datasets presented in this study can be found in online repositories. The names of the repository/repositories and accession number(s) can be found below: the joint CCDC/FIZ Karlsruhe online deposition service: https://www.ccdc.cam.ac.uk/structures/; the deposition number CSD-2280897.
