# Supplementary material for: The synthesis of novel lanthanum hydroxyborate at extreme conditions
Source: Front Chem. 2023 Sep 28;11:1259000. doi: 10.3389/fchem.2023.1259000 (PMC10568730; doi:10.3389/fchem.2023.1259000)
Supplement: Supplementary file 1 [file DataSheet1.PDF]

Supplementary materials

The synthesis of novel lanthanum hydroxyborate at extreme conditions

Olga Ibragimova*[1], Lia Vaquero[1], Zain Hussein[2], Vadim Drozd[3], Stella Chariton[4], Vitali Prakapenka[4], Irina Chuvashova*[1,2].

[1]Department of Chemistry and Biochemistry, Florida International University, Miami, Florida, USA
[2]Physics Department, Florida International University, Miami, Florida, USA
[3]Department of Mechanical and Materials Engineering, Florida International University, Miami, Florida, USA
[4]Center for Advanced Radiation Sources, University of Chicago, Chicago, Illinois, U.S.A

*** Correspondence:**
Irina Chuvashova
irina.chuvashova@fiu.edu
Olga Ibragimova
oibra005@fiu.edu

Keywords: high pressure, high temperature, synthesis, nonlinear optics, birefringence, borates, rare earth borates, lanthanum

List of tables and figures:

Table S1. Atomic coordinates and isotropic equivalent displacement parameters U_{eq} [Å^2] (for B-atom only) for $La_2B_2O_5(OH)_2$.

Table S2. Anisotropic displacement parameters U_{ij} [Å^2] for $La_2B_2O_5(OH)_2$.

Table S3. Interatomic distances [Å] obtained with atomic parameters refinement (standard deviations in parentheses) in $La_2B_2O_5(OH)_2$.

Figure S1. EDS spectra of starting mixture with SEM image of the reaction mixture before loading in a DAC (inset).

Figure S2. Coordination spheres for three crystallographically different lanthanum atoms: (A) La01 coordinated by 10 oxygens, (B) La02 coordinated by 12 oxygens, (C) La03 coordinated by 9 oxygens.

Figure S3. The fragment of the structure with all three lanthanum atoms linked with the BO_3 group. The bond distances [Å] for B-O are shown in black.

Figure S4. The electronic band structure calculated with GGA method: (A) for ambient pressure, and (B) for 30 GPa.

Figure S5. (A) The positions of BO_3 groups within the unit cell from the perspective of the space group diagram. The boron triangles are shown in different colors depending on the plane. (B) The position of BO_3 groups in unit cell along C axis.

Table S1. Atomic coordinates and isotropic equivalent displacement parameters U_{eq} [Å^2] (for B-atom only) for $La_2B_2O_5(OH)_2$.

Atom	Wyckoff Position	*x*	*y*	*z*	U_{eq}
La01	*4d*	0.33333	0.66667	0.56794(10)	-
La02	*2b*	0	0	0.5	-
La03	*6f*	0.34703(19)	0.34703(19)	0.75	-
O01	*4d*	0.33333	0.66667	0.69797(13)	-
O02	*12g*	-0.01652(17)	0.23830(17)	0.39262(7)	-
O03	*12g*	0.31874(19)	0.40938(17)	0.45986(7)	-
O04	*2a*	0	0	0.75	-
O05	*12g*	0.67269(19)	0.57946(19)	0.6661(7)	-
B01	*12g*	0.78518(2)	0.631(2)	0.6038(9)	0.003(3)

Table S2. Anisotropic displacement parameters U_{ij} [Å^2] for $La_2B_2O_5(OH)_2$.

Atom	U_{11}	U_{22}	U_{33}	U_{12}	U_{13}	U_{23}
La01	0.0097(4)	0.0097(4)	0.0154(10)	0.0048(2)	0	0
La02	0.0103(6)	0.0103(6)	0.0183(15	0.0052(3)	0	0
La03	0.0099(4)	0.0099(4)	0.0155(7)	0.0039(5)	0.0013(3)	- 0.0013(3)
O01	0.008(5)	0.008(5)	0.068(18)	0.004(2)	0	0
O02	0.016(5)	0.020(5)	0.004(7)	0.010(5)	0.003(4)	- 0.001(4)
O03	0.018(5)	0.010(5)	0.012(8)	0.008(4)	- 0.002(4)	0.000(4)
O04	0.003(7)	0.003(7)	0.13(3)	0.002(4)	0	0
O05	0.013(5)	0.030(7)	0.019(9)	0.010(5)	- 0.002(4)	0.008(5)

Table S3. Interatomic distances [Å] obtained with atomic parameters refinement (standard deviations in parentheses) in $La_2B_2O_5(OH)_2$.

La01-O01	2.28(2)	La02-O02	2.478(11)	La03-O01	2.326(9)	O02-O03	2.723(17)
La01-O02	2.542(13)		2.478(14)		2.326(9)	O02-O05	2.208(15)
	2.542(16)	La02-O03	2.541(9)	La03-O02	2.583(12)		2.653(14)
	2.542(12)		2.541(12)		2.583(12)		2.763(17)
La01-O03	2.502(12)		2.541(16)	La03-O04	2.275(3)	O02-B01	1.321(15)
	2.502(11)		2.541(9)	La03-O05	2.404(11)		2.454(16)
	2.502(15)		2.541(12)		2.440(17)	O03-O03	2.493(16)
	2.613(14)		2.541(16)		2.404(11)	O03-O05	2.202(17)
	2.613(17)	La02-B01	2.780(14)		2.440(17)	O03-B01	1.259(19)
	2.613(12)		2.780(15)	O01-O05	2.619(15)		2.783(18)
La02-O02	2.478(12)		2.780(19)		2.619(18)	O05-O05	2.761(19)
	2.478(11)		2.780(14)		2.619(13)		2.76(2)
	2.478(14)		2.780(15)	O02-O03	2.236(15)	O05-B01	1.26(2)
	2.478(12)		2.780(19)		2.666(13)		2.715(18)

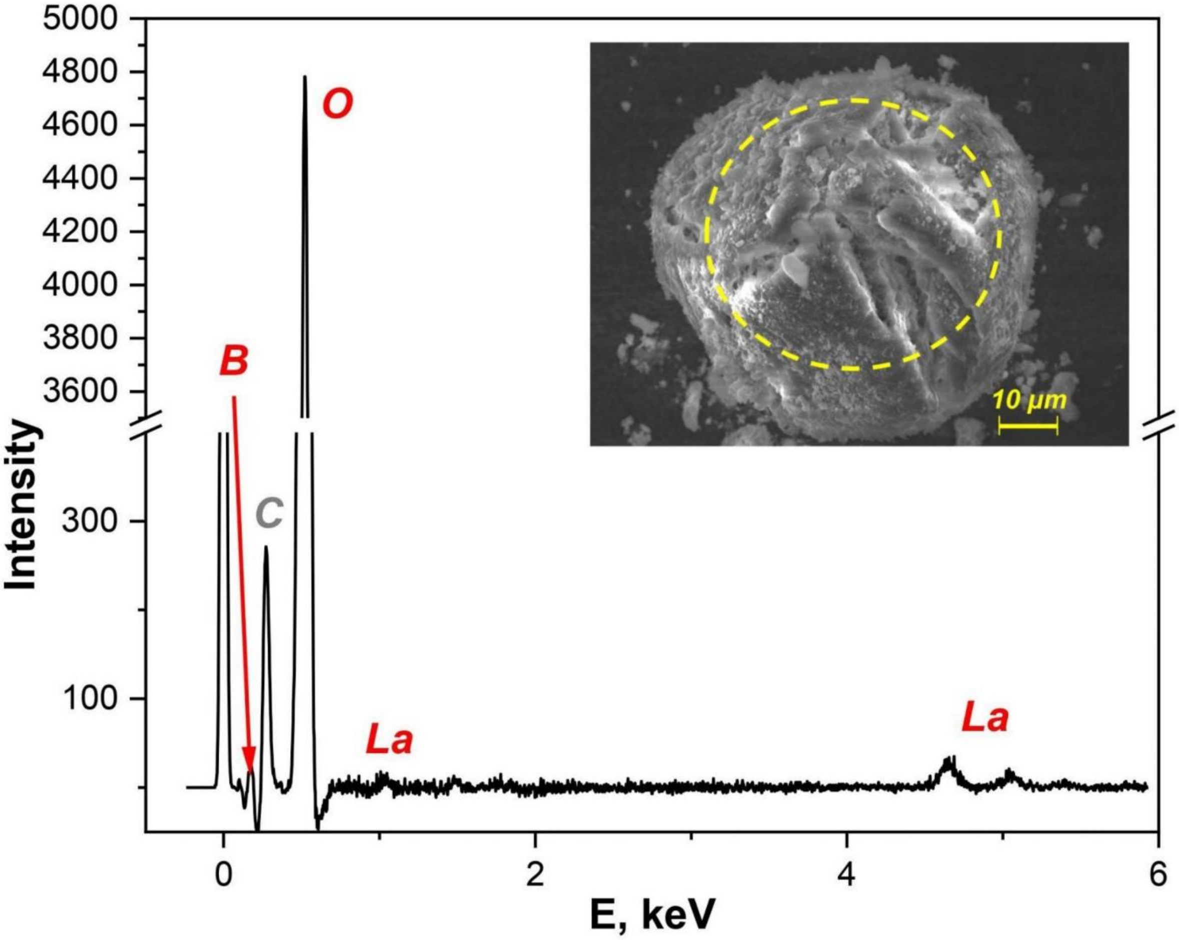

Figure S1. EDS spectra with SEM image as an insert of starting mixture before loading in a DAC.

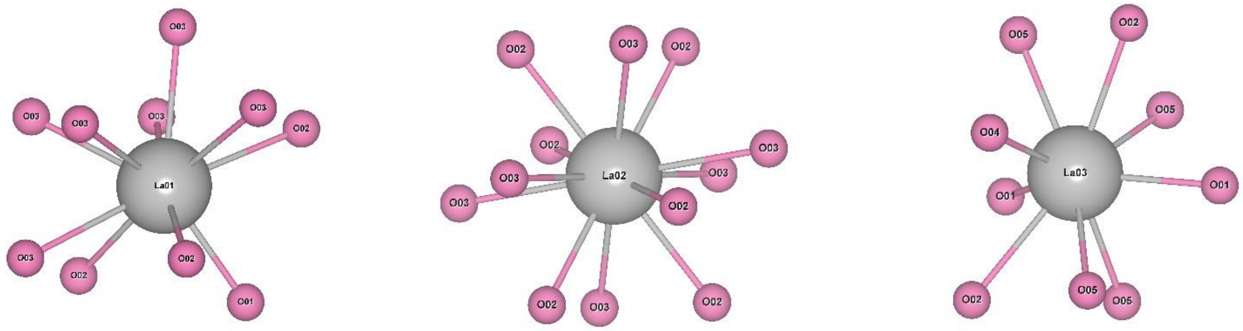

(A) **(B)** **(C)**

Figure S2. Coordination spheres for three crystallographically different lanthanum atoms: (A) La01 coordinated by 10 oxygens, (B) La02 coordinated by 12 oxygens, (C) La03 coordinated by 9 oxygens.

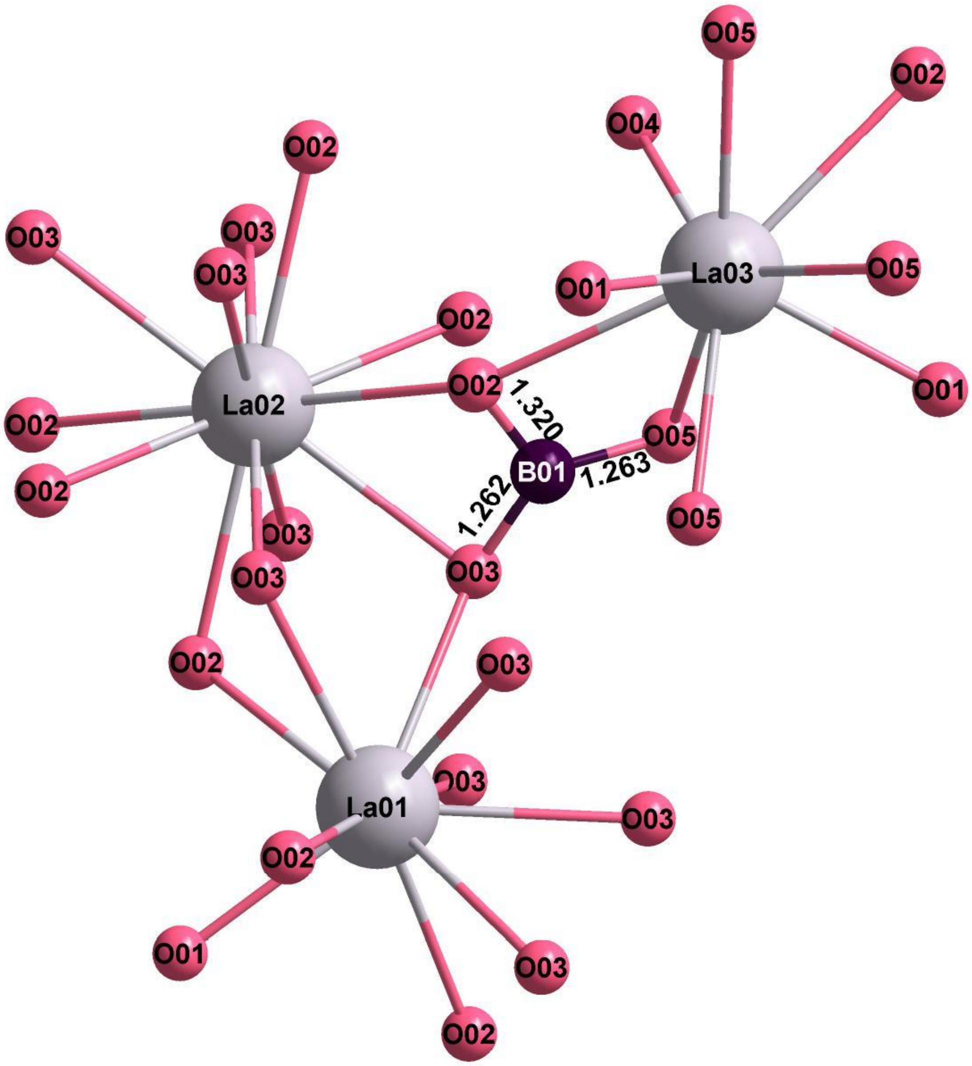

Figure S3. The fragment of the structure with all three lanthanum atoms linked with the BO_3 group. The bond distances [Å] for B-O are shown in black.

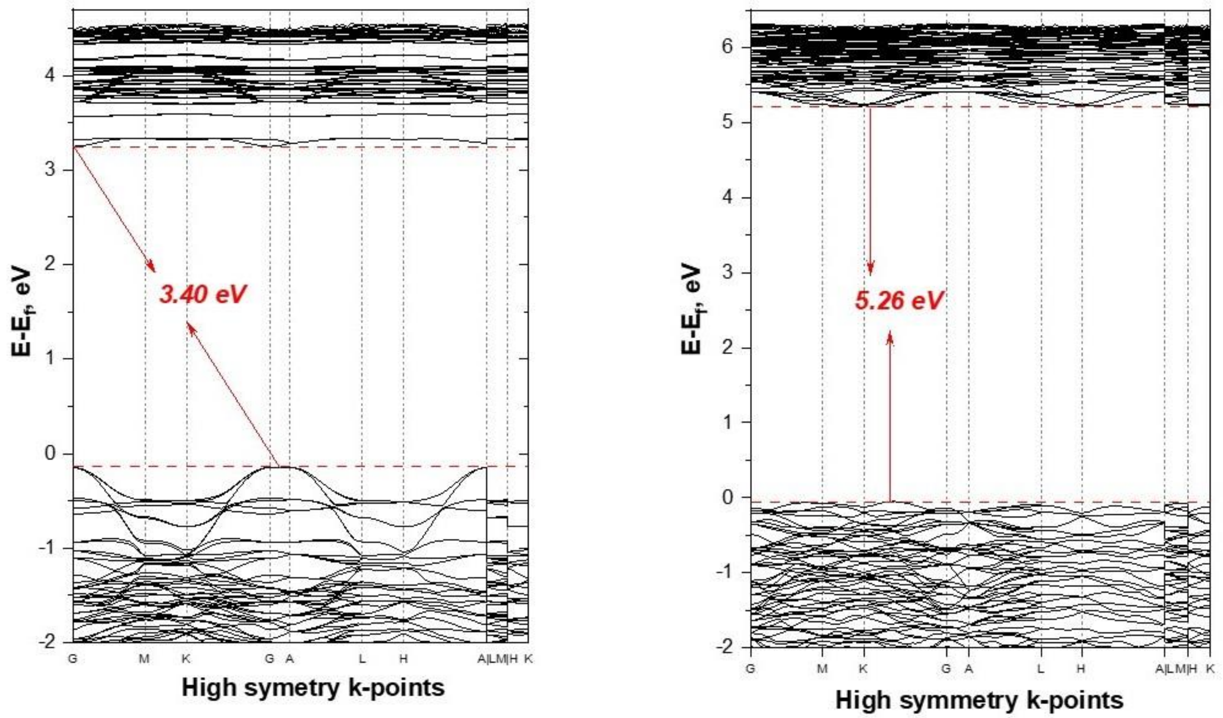

(A) **(B)**

Figure S4. The electronic band structure calculated with GGA method: (A) for ambient pressure, and (B) for 30 GPa.

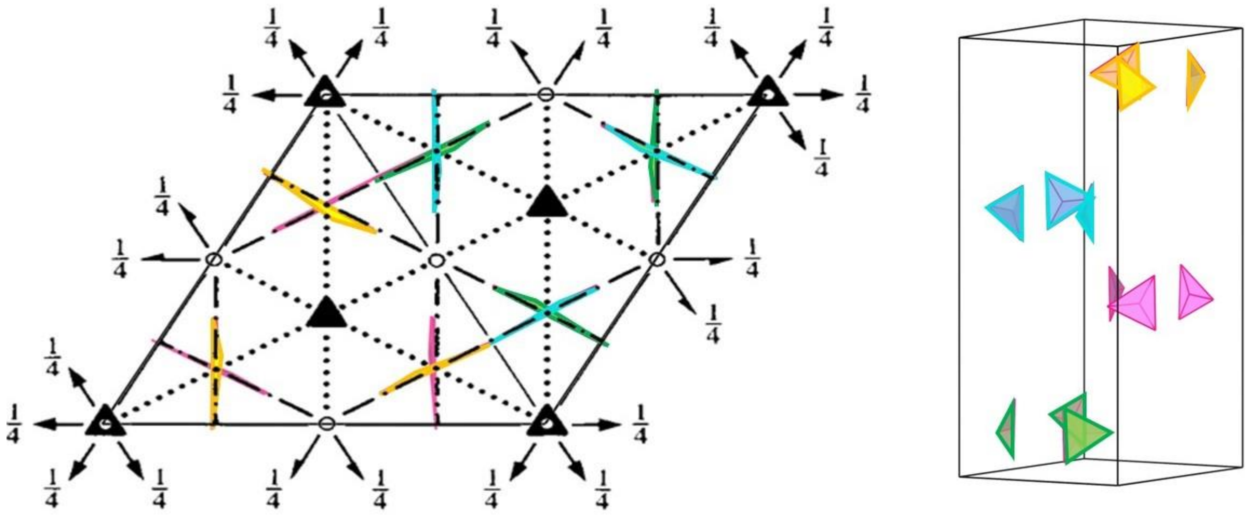

(A) **(B)**

Figure S5. (A) The positions of BO_3 groups within the unit cell from the perspective of the space group diagram. The boron triangles are shown in different colors depending on the plane. (B) The position of BO_3 groups in unit cell along C axis.The z distance between the layers of the triangulars marked in different color is 1/4.
